# Supplementary material for: Ventromedial prefrontal cortex compression during concept learning
Source: Nat Commun. 2020 Jan 7;11:46. doi: 10.1038/s41467-019-13930-8 (PMC6946809; doi:10.1038/s41467-019-13930-8)
Supplement: Supplementary file 1 — Supplementary Information [file 41467_2019_13930_MOESM1_ESM.pdf]

Supplementary Information

Ventromedial prefrontal cortex compression during concept learning

Mack et al.

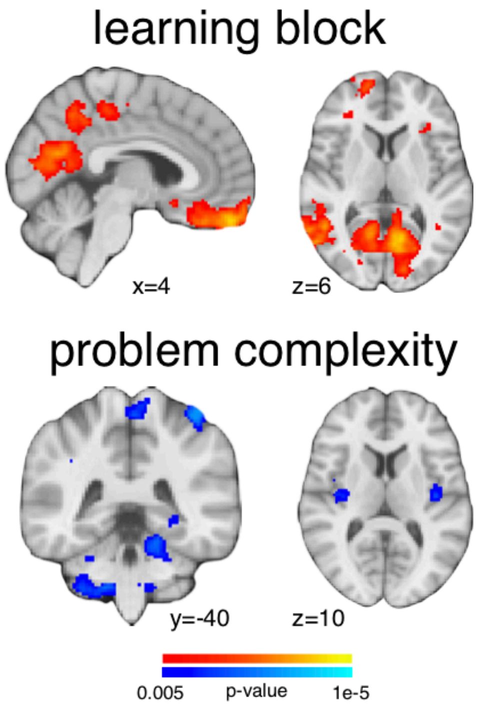

Supplementary Figure 1: Whole brain maps of neural compression analysis for main effects of learning block and problem complexity (N=23). Neural compression increased with learning block (top) in several regions including vmPFC, posterior cingulate, and retrosplenial cortex. Neural compression decreased with higher problem complexity (bottom) in bilateral insula, superior parietal, and cerebellum (see Supplementary Table 1 for all identified clusters). These maps were generated with a voxel-wise threshold of p = 0.005 and cluster-extent threshold of p = 0.05.

	peak location	peak t value	size	labels
learning block	30, -78, 38	4.56	20162	occipital, inferior parietal, angular gyrus
	42, 22, -42	4.92	10450	bilateral anterior temporal, ventromedial prefrontal cortex
	-18, 28, 42	3.62	671	left superior frontal gyrus
	22, -88, -32	3.81	244	right cerebellum
problem complexity	-14, -22, -34	3.7	1462	cerebellum, brain stem
	34, -14, 14	3.33	744	right insula
	-42, -42, 62	3.9	537	left superior parietal
	-2, -30, 66	3.48	524	bilateral precentral gyrus
	-40, -14, 6	3.4	411	left insula

Supplementary Table 1: Significant clusters showing main effects of learning block or problem complexity from the neural compression searchlight analysis. Cluster information includes the peak voxel location in MNI coordinates (x, y, z), the t statistic of the peak voxel, the cluster size in number of voxels, and the corresponding anatomical label(s) as defined in the Harvard-Oxford Structural Atlas.
