# Supplementary material for: Ventromedial prefrontal cortex compression during concept learning
Source: Nat Commun. 2020 Jan 7;11:46. doi: 10.1038/s41467-019-13930-8 (PMC6946809; doi:10.1038/s41467-019-13930-8)
Supplement: Supplementary file 3 — Reporting Summary [file 41467_2019_13930_MOESM3_ESM.pdf]

nature research

Corresponding author(s): Michael L. Mack

Last updated by author(s): May 21, 2019

Reporting Summary

Nature Research wishes to improve the reproducibility of the work that we publish. This form provides structure for consistency and transparency in reporting. For further information on Nature Research policies, see Authors & Referees and the Editorial Policy Checklist .

Statistics

For all statistical analyses, confirm that the following items are present in the figure legend, table legend, main text, or Methods section.

n/a	Confirmed	
☐	☒	The exact sample size (*n*) for each experimental group/condition, given as a discrete number and unit of measurement
☐	☒	A statement on whether measurements were taken from distinct samples or whether the same sample was measured repeatedly
☐	☒	The statistical test(s) used AND whether they are one- or two-sided<br>*Only common tests should be described solely by name; describe more complex techniques in the Methods section.*
☐	☒	A description of all covariates tested
☐	☒	A description of any assumptions or corrections, such as tests of normality and adjustment for multiple comparisons
☐	☒	A full description of the statistical parameters including central tendency (e.g. means) or other basic estimates (e.g. regression coefficient) AND variation (e.g. standard deviation) or associated estimates of uncertainty (e.g. confidence intervals)
☐	☒	For null hypothesis testing, the test statistic (e.g. *F*, *t*, *r*) with confidence intervals, effect sizes, degrees of freedom and *P* value noted<br>*Give P values as exact values whenever suitable.*
☐	☒	For Bayesian analysis, information on the choice of priors and Markov chain Monte Carlo settings
☐	☒	For hierarchical and complex designs, identification of the appropriate level for tests and full reporting of outcomes
☐	☒	Estimates of effect sizes (e.g. Cohen's *d*, Pearson's *r*), indicating how they were calculated

Our web collection on statistics for biologists contains articles on many of the points above.

Software and code

Policy information about availability of computer code

Data collection	Behavioural data was collected with Matlab (2014b) and Psychtoolbox (3.0.9) running on OS X (10.7)
Data analysis	Behavioural data analysis and SUSTAIN model fits were run using custom python 2.7 scripts. MRI data preprocessing and analyses were conducted with FSL 6.0, Advanced Normalization Tools (1.9), AFNI 3dClustSim (16.3.12), custom python 2.7 scripts (libraries: pymvpa 2.6, scikit-learn 0.17.1, statsmodels 0.8), and custom R scripts (libraries: rstanarm 2.18.2).

For manuscripts utilizing custom algorithms or software that are central to the research but not yet described in published literature, software must be made available to editors/reviewers. We strongly encourage code deposition in a community repository (e.g. GitHub). See the Nature Research guidelines for submitting code & software for further information.

Data

Policy information about availability of data

All manuscripts must include a data availability statement. This statement should provide the following information, where applicable:

- Accession codes, unique identifiers, or web links for publicly available datasets
- A list of figures that have associated raw data
- A description of any restrictions on data availability

The data collected for this study are available for download: https://osf.io/5byhb/.

Field-specific reporting

Please select the one below that is the best fit for your research. If you are not sure, read the appropriate sections before making your selection.

Behavioural & social sciences study design

All studies must disclose on these points even when the disclosure is negative.

Study description	Quantitative experimental study.
Research sample	23 University of Texas at Austin undergraduate students (11 females, mean age 22.3 years, age range 18-31). All subjects were right handed and had normal or corrected-to-normal vision.
Sampling strategy	Sample size was determined by a power analysis on findings from a previous study employing similar fMRI pattern similarity methods (Mack, Preston, & Love, 2013). We examined the effect size (dz = 0.97) of a paired comparison between neural similarity in inferior frontal gyrus to two computational model-derived similarity matrices. With alpha = 0.05 and beta = 0.8, the required sample size to detect this effect is 11 participants. Based on our previous experience with functional imaging of the whole brain, as well as with category learning experiments, we doubled the minimum sample size to 22 participants.
Data collection	Data was collected at the University of Texas at Austin Imaging Research Center using a Siemens Skyra 3T MRI scanner. Stimulus timing and behavioural data collection was computer based. Two researchers, both of whom where not blind to the study hypotheses, were present during data collection.
Timing	March 16, 2014 - December 12, 2014
Data exclusions	No data were excluded from analysis.
Non-participation	No participants dropped out/declined to participate.
Randomization	Participants were not allocated into different experimental groups.

Reporting for specific materials, systems and methods

We require information from authors about some types of materials, experimental systems and methods used in many studies. Here, indicate whether each material, system or method listed is relevant to your study. If you are not sure if a list item applies to your research, read the appropriate section before selecting a response.

Materials & experimental systems

n/a	Involved in the study
☒	☐ Antibodies
☒	☐ Eukaryotic cell lines
☒	☐ Palaeontology
☒	☐ Animals and other organisms
☐	☒ Human research participants
☒	☐ Clinical data

Methods

n/a	Involved in the study
☒	☐ ChIP-seq
☒	☐ Flow cytometry
☐	☒ MRI-based neuroimaging

Human research participants

Policy information about studies involving human research participants

Population characteristics	See above.
Recruitment	Participants were recruited from the University of Texas at Austin student population and surrounding Austin, Texas community. There were no apparent biases in recruitment that would impact the current findings.
Ethics oversight	University of Texas at Austin Institutional Review Board

Note that full information on the approval of the study protocol must also be provided in the manuscript.

Magnetic resonance imaging

Experimental design

Design type	Event-related design.
Design specifications	Each participant completed 12 blocks of 32 trials. Each block was 388 seconds. The entire experiment lasted approximately 65 minutes.

Behavioral performance measures	Correct and incorrect responses along with response times were collected. The likelihood of correct responses over learning was assessed with a linear mixed-effect regression model to confirm that participants were engaged in the task.

Acquisition

Imaging type(s)	functional and structural
Field strength	3T
Sequence & imaging parameters	A high-resolution T1-weighted MPRAGE structural volume (TR = 1.9s, TE = 2.43ms, flip angle = 9°, FOV = 256mm, matrix = 256x256, voxel dimensions = 1mm isotropic) was acquired for coregistration and parcellation. Two oblique coronal T2-weighted structural images were acquired perpendicular to the main axis of the hippocampus (TR = 13,150ms, TE = 82ms, matrix = 384x384, 0.4x0.4mm in-plane resolution, 1.5mm thru-plane resolution, 60 slices, no gap). High-resolution functional images were acquired using a T2*-weighted multiband accelerated EPI pulse sequence (TR = 2s, TE = 31ms, flip angle = 73°, FOV = 220mm, matrix = 128x128, slice thickness = 1.7mm, number of slices = 72, multiband factor = 3) allowing for whole brain coverage with 1.7mm isotropic voxels.
Area of acquisition	Whole brain

Diffusion MRI ☐ Used ☒ Not used

Preprocessing

Preprocessing software	Tools from FSL 5.0.9 was used for preprocessing including mcflirt, fslmaths, and bet2. Volumes were high-pass filtered (128s), detrended to remove linear trends within each run, and spatially smoothed with a 3mm FWHM Gaussian kernel.
Normalization	ANTs 1.9 was used for normalization.
Normalization template	MNI152 2mm T1
Noise and artifact removal	Motion correction within each run was performed with mcflirt. The resulting 6 motion parameters were included as covariates in the single-trial GLM analysis.
Volume censoring	No volume censoring was performed.

Statistical modeling & inference

Model type and settings	fMRI data was first submitted to mass univariate GLMs for single-trial parameter estimates. This was performed separately for each subject, learning block, and problem. The LS-S method was employed and covariates included motion parameters, feedback events, and response events in addition to the onsets of each trial. The resulting single-trial estimates were then submitted to a novel MVPA that calculated PCA across trials. Both of these analyses can be considered fixed effects. Finally, a linear mixed effect regression was conducted on the PCA results at each voxel location with participants as a random factor.
Effect(s) tested	The central effect was the degree that the dimensionality of neural representations changed over learning and how this change varied across problem complexity. Learning block and problem complexity were fully crossed and were within-participant factors.

Specify type of analysis: ☒ Whole brain ☐ ROI-based ☐ Both

Statistic type for inference (See Eklund et al. 2016)	Cluster-wide inference was performed with a voxelwise threshold of p = 0.001 and cluster correction at p = 0.05. This resulted in a cluster extent threshold of 259 voxels.
Correction	Multiple comparison correction for whole brain analyses were conducted using 3dClustSim using the acf option, second nearest neighbor clustering, and 2-sided thresholding. ACF data smoothness was calculated from the residuals of the single-trial GLM estimates for each subject and then averaged for the 3dClustSim calculations. This correction ensured a family wise error rate of alpha = 0.05.

Models & analysis

n/a	Involved in the study
☒	☐ Functional and/or effective connectivity
☒	☐ Graph analysis
☐	☒ Multivariate modeling or predictive analysis

Multivariate modeling and predictive analysis	PCA was conducted on single-trial GLM parameter estimates across voxels within searchlight spheres with a radius of 4 voxels. Within each voxel, the resulting dimensionality values were analyzed with a linear mixed effects regression model with learning block, problem complexity, behavioural accuracy and learning problem order as covariates. The t-statistics for the main effects of these covariates as well as the interaction of learning block and problem complexity were saved to separate statistical brain maps.
